# Supplementary material for: Patient and Clinician Preferences for Genetic and Genomic Testing in Non-Small Cell Lung Cancer: A Discrete Choice Experiment
Source: J Pers Med. 2022 May 26;12(6):879. doi: 10.3390/jpm12060879 (PMC9225087; doi:10.3390/jpm12060879)
Supplement: Supplementary file 1 [file jpm-12-00879-s001.zip › jpm-1686035-supplementary.pdf]

SUPPLEMENTARY MATERIALS

Table S1. Attributes Identified During Qualitative Interviews

Attribute	Patients	Physicians	Pathologists
Accuracy	Y	?	Y
Type of biopsy	Y	Y	Y
Type of test	N	N	N
Size of panel	Y	Y	Y
Reproducibility	Y	Y	Y
Test turnaround time	Y	Y	Y
Risks involved	?	Y	?
Cost	Y	Y	?
Location of test (overseas vs Australia)	?	?	N
Reporting	-	Y	Y

Table S2. Preference share scenario analyses

	WES/WGS	CGP	Medium gene panel	Small gene panel
		**Patient preference share**		
**Number of genes**	>500 genes	80-500 genes	20-80 genes	5-20 genes
**Chance of actionable outcome**	75% [41]	50%	25%	25%
**Funding of treatment**	Funded	Funded	Funded	Funded
**Tissue requirements**	No additional procedures required	No additional procedures required	No additional procedures required	No additional procedures required
**Turnaround**	6-12 weeks [42,43]	2-4 weeks*	2-4 weeks	<2 weeks
**Processing and analysis**	Australia	Australia	Australia	Australia
**Germline findings**	Yes	Yes	No	No
**Cost**	$5000 [44]	$3000	$800†	$400 [44]
**Proportion preferring new test**	91.3%	91.26%	58.69%	44.9%
		**Physician preference share**		
**Number of genes**	>500 genes	80-500 genes	20-80 gene	5-20 genes
**Chance of actionable outcome**	75%	50%	25%	25%

	WES/WGS	CGP	Medium gene panel	Small gene panel
**Funding of treatment**	Funded	Funded	Funded	Funded
**Tissue requirements**	No additional procedures required	No additional procedures required	No additional procedures required	Re-biopsy
**Turnaround time**	6-12 weeks	2-4 weeks*	2-4 weeks	<2 weeks
**Processing and analysis**	Australia	Australia	Australia	Australia
**Germline findings**	Yes	Yes	No	No
**Cost**	$5000[44]	$3000	$800†	$400 [44]
**Interpretation and reporting**	Mutation status and established treatments	Mutation status and established treatments	Mutation status and established treatments	Mutation status and established treatments
**Proportion preferring new test**	96.88%	97.17%	68.41%	11.48%

** based on turnaround times at the time of writing for Foundation One® CDx assay (F1CDx) as part of the MoST and ASPiRATION studies*
† based on Medicare Benefits Schedule (MBS) item 73376 of 4 or more genes for select cancers capped at $800
Abbreviations: CGP = comprehensive genomic profiling; WES = whole exome sequencing; WGS = whole genome sequencing

Assumptions of each type of test

Table S3. *Base Case*

BASE CASE	**Current standard diagnostic pathway**	**WES/WGS**	**CGP**	**Medium gene panel**
Number of genes	<5 genes; sequential	>500 genes	80-500 genes	20-80 genes
Chance of actionable outcome	25%	75%*	50%	25%
Funding of treatment				Funded
Tissue requirements				
Turnaround time	<2 weeks	6-12 weeks	2-4 weeks	2-4 weeks
Processing and analysis	Australia	Australia	Australia	Australia
Germline findings	No	Yes	Yes	No
Reporting	Reports approved treatments only	Reports all treatments (incl. trials etc.)	Reports all treatments (incl. trials etc.)	Reports approved treatments only
Cost	$0	$5000	$3000	$800

Table S4. Patients

Patients	WES/WGS	WES/WGS	CGP	CGP	Medium gene panel
Funding of treatment	Funded	Not Funded	Funded	Not Funded	Funded
Tissue requirements					
**Proportion preferring new test if:**					
INPUT: Tissue requirements = none	91.30%	71.97%	91.26%	91.84%	58.69%
INPUT: Tissue requirements = tumour re-biopsy	77.52%	45.75%	77.43%	78.7%	31.81%
INPUT: Tissue requirements = blood test	85.91%	59.86%	85.85%	86.73%	45.21%

Table S5. Physicians

Physicians	WES/WGS	WES/WGS	CGP	CGP	Medium gene panel
Funding of treatment	Funded	Not Funded	Funded	Not Funded	Funded
Tissue requirements					
**Proportion preferring new test if:**					
INPUT: Tissue requirements = none	97.63%	74.66%	97.86%	80.83%	68.41%
INPUT: Tissue requirements = tumour re-biopsy	86.11%	30.70%	87.28%	38.8%	24.56%
INPUT: Tissue requirements = blood test	95.45%	59.98%	95.87%	68.19%	52.41%

Supplementary document S1: Value Mapping of Gene Alteration Status Testing Options in Non Small Cell Lung Cancer Among Patients and Clinicians- **Qualitative Pathologist Discussion Guide**

**Research Objectives (for interviewer reference):**
The aim of this study is to develop a greater understanding of the key attributes / features that pathologists consider important with gene alteration status test for locally advanced (stage IIIb) and metastatic (stage IV) Non Small Cell Lung Cancer (NSCLC).<br><br>The specific research objectives for this qualitative phase are to enhance understanding of:<br><br>1. Pathologists perceptions of gene alteration status testing within locally advanced (stage IIIb) and metastatic (stage IV) NSCLC patients.<br>2. What is the pathologist role within gene alteration status testing?<br>3. Key attributes of gene alteration status testing that pathologists value and the relative importance of these attributes (these attributes will be used in the design of the quantitative phase of research).<br>4. What may drive uptake of these tests<br><br><u>FOR INTERVIEWER REFERENCE:</u><br>**For this research, tests that fall within the scope of 'gene alteration status testing' include:**<br>1) Any assays that detect somatic alterations in solid tissue or liquid biopsy [e.g. detection of circulating DNA (cDNA) or circulating tumour DNA (ctDNA)]; that is alterations that occur spontaneously over the course of a person's lifetime or through interaction with known carcinogens.<br>2) Any standard diagnostic (e.g. IHC/FISH) detecting qualitative sequence level changes (e.g. EGFR mutations variants or alterations,) or quantitative expression level (protein) biomarkers such as (e.g. PD-L1)<br>3) Any large or small gene panel using next-generation sequencing (NGS) technology with an assay based on PCR or amplicon (e.g. Hotspot) or hybrid-capture [e.g. Comprehensive genomic profiling (CGP)] target enrichment.<br>4) Whole exome or whole genome sequencing performed on DNA extracted from tumour tissue.<br><br>**For this research, tests that fall outside the scope of 'gene alteration status testing' include:**<br>1) Any assay that detects germline alterations; that is alterations that are inherited and occur in families (e.g. BRCA1 and BRCA2). These tests would be done in DNA extracted white blood cells in peripheral whole blood and a genetic counsellor would be involved to counsel the patient and their family members.

**Discussion Guide**		
Aim		**Discussion**

To explain purpose of research	1.	**Warm Up and Introduction** (2 min)<br>● *Thank respondent for taking part in the research.*<br>● *Explain purpose of the research being conducted:* [We are conducting research in the area of locally advanced (stage IIIb) and metastatic (stage IV) NSCLC. I am interested to learn about your role in the management of this condition and what your perceptions are regarding gene alteration status testing for such patients.<br>● *Explain anonymity:* Participants are guaranteed under Privacy Act and AMSRS code / findings from interviews will not be linked back to individual people.<br>● *Explain obligation to report Adverse Events (AE):* Although all responses will, of course, be treated in confidence, should you raise an adverse event experienced by your patients after using one of our client's products or medications, we will need to report this, so that they can keep the knowledge of the safety profile of their medicines up to date. This also applies if the event has already been reported by you directly to the company or the regulatory authority CONFIRM THAT PATHOLOGIST HAS READ AND SIGNED CONSENT FORM<br>● *Ask permission to record (for report-writing purposes).*
Gain background information on participant and on locally advanced (stage IIIb) and metastatic (stage IV) NSCLC workload.	2.	**Participant Profile** (3 min)<br>Can I begin by asking you about your work situation?<br>● Where is your key place of work?<br>○ Do you work in both private / public settings?<br>○ Location? (Rural, regional, metro?)<br>○ Socio-economic area?<br>○ Are you part of a Multi-Disciplinary Team (MDT)? Which?<br>● Can you please summarise what your key duties / responsibilities are as a pathologist?<br>○ Do you have any special interests or areas of focus within pathology?<br>● Thinking now about locally advanced (stage IIIb) and metastatic (stage IV) NSCLC, approximately what proportion of your overall pathology workload is for this condition?
Determine pathologist's perceptions of gene alteration status testing – gain feedback on standard vs. CGP tests	3.	**Pathologist Perceptions of Gene Alteration Status Testing** (20 min)<br>I would now like to discuss gene alteration status testing and its role within locally advanced (stage IIIb) and metastatic (stage IV) NSCLC.<br>Before we begin with this, I would like to mention that for this research, tests that fall within the scope of 'gene alteration status testing' include:<br>1) Any assays that detect somatic alterations in solid tissue or liquid biopsy [e.g. detection of circulating DNA (cDNA) or circulating tumour DNA (ctDNA)]; that is alterations that occur spontaneously over the course of a person's lifetime or through interaction with known carcinogens.

		2) Any standard diagnostic (e.g. IHC/FISH) detecting qualitative sequence level changes (e.g. EGFR mutations variants or alterations,) or quantitative expression level (protein) biomarkers such as (e.g. PD-L1)<br>3) Any large or small gene panel using next-generation sequencing (NGS) technology with an assay based on PCR or amplicon (e.g. Hotspot) or hybrid-capture [e.g. Comprehensive genomic profiling (CGP)] target enrichment.<br>4) Whole exome or whole genome sequencing performed on DNA extracted from tumour tissue.<br><br>Tests that fall outside the scope of 'gene alteration status testing' include:<br><br>1) Any assay that detects germline alterations; that is alterations that are inherited and occur in families (e.g. BRCA1 and BRCA2). These tests would be done in DNA extracted white blood cells in peripheral whole blood and a genetic counsellor would be involved to counsel the patient and their family members.<br><br>As some background information:<br><br><u>Terminology Used</u><br><br>● What terminology do you personally use when referring to such tests?<br>o When talking to other HCPs?<br>o If talking to patients?<br>● Does terminology vary depending on what type of test is ordered? How so?<br><br><u>FOR REMAINDER OF INTERVIEW, USE PARTICPANT'S PREFERRED TERMINOLOGY</u><br><br><u>General Perceptions of gene alteration status testing</u><br><br>● What do you believe is the key role of gene alteration status testing for locally advanced (stage IIIb) and metastatic (stage IV) NSCLC?<br>o What are the reasons gene alteration status tests are used in this therapy area?<br>● What are the positives with testing? How can patients benefit?<br>● What are challenges? <u>POTENTIAL PROBES:</u><br>o Biopsy sampling? (e.g. limited tumour tissue? Preservation of tissue?)<br>▪ Thoughts on tissue vs. liquid biopsies? Which do you prefer and why?<br>o Accuracy of test?<br>▪ How do you assess accuracy? Do you have a certain standard or minimum accuracy value?<br>▪ Can you tell me a little about 'false negatives' and 'false positives'? How does this impact on patients?<br>o Interpretation of results? Who has the key responsibility for this? Yourself? Oncologists? Other HCP?

		- Turn around for test results? How so? What does this mean for patients?<br>- A general lack of testing awareness and understanding?<br>- Among HCPs? Among patients?<br>- Risks involved? What type of risks?<br>- Legislation issues?<br>- Patient consent? How so?<br><br><u>Preferred testing method for gene alteration status</u><br><br>Can you tell me about the tissue you prefer to process for gene alteration status testing?<br><br>● Initial biopsy?<br>○ I.e. biopsy from first diagnosis / at determination of locally advanced (stage IIIb) and metastatic (stage IV) disease?<br>● Re-biopsy?<br>○ Biopsy from primary tumour tissue?<br>○ Biopsy from potential metastases?<br>● Are you aware of what proportion of patients require re-biopsy due to insufficient sampling of initial biopsy?<br>● At what point would you do a re-biopsy (initially or later in pathway)<br>● What are your thoughts on having to re-biopsy a patient?<br><br><u>Differences existing with gene alteration status testing within various settings</u><br><br>And now, just a few questions regarding use of gene alteration status testing in different areas or settings within locally advanced (stage IIIb) and metastatic (stage IV) NSCLC.<br><br>● To what extent are there differences in use of gene alteration status tests between states or in metro vs. regional and rural areas (if any)?<br>○ How does this impact on patient management in metro vs. regional and rural areas?<br>● To what extent are there differences in the private vs. public setting (if any)?<br>○ How does this impact on patient management in private vs. public setting?<br><br>I'd now like to ask you about different gene alteration status tests used for locally advanced (stage IIIb) and metastatic (stage IV) NSCLC.<br><br><u>Feedback on conventional / standard gene alteration status algorithm</u>

- Can you please take me through the standard gene alteration status testing algorithm for this condition? I.e. sequential single marker genetic tests? PROBE ON:
    - What tests are used and why? (e.g. EGFR, ALK, ROS?)
    - How are these tests used?
        - At what point in the patient pathway are they ordered (i.e. at diagnosis of advanced / metastatic disease? during treatment journey?)
        - If you are aware, how often are you processing these tests for an individual patient?
    - How do these tests guide treatment decisions?
    - What are your overall thoughts about standard testing?
        - Advantages - what type of concrete improvements do they provide for patients?
        - Disadvantages? Limitations?

Feedback on advancements in Gene Alteration Status Testing

- Can you now tell me about newer / more advanced gene alteration status testing options? I.e. Genomic Profiling?
    - Next Generation Sequencing (NGS)
    - Hot Spot Panels
    - Comprehensive Genomic Profiling (CGP)
    - Other?
- Overall, what are your thoughts and feelings on the direction of genomic profiling in this cancer space?
    - What implications does this have within your pathology practice (if any)?

Feedback on Comprehensive Genomic Profiling (CGP)

I'd now like to focus a little more on CGP.

- To what extent are you processing CGP tests for this condition? IF PROCESSING CGP ASK:
    - What proportion of these are via tumour tissue vs. liquid biopsy panels?
- In what situations are these tests being ordered (as opposed to standard tests?). I.e. are you aware of the reasons why the ordering physician has selected CGP for a patient?
    - E.g. Patient requests? Patient factors? For additional insights?
- At what point in the patient pathway are these tests being ordered? (i.e. before 1st-line treatment? Progressive disease and lack of patient response? FOR PROGRESSIVE DISEASE / LACK OF PATIENT RESPONSE
    - Would you have preferred to do this upfront after diagnosis? Why do you say that?

<table>
<tr>
<td></td>
<td></td>
<td>

If you are aware, how often are you processing these tests for an individual patient?
How do these tests guide treatment decisions? How do they differ in this regard compared to standard tests?
How may a treatment change as a result of CGP tests, if at all?
Can you provide examples of the specific CGP tests you are processing?

Lab developed tests / in house testing?
Send out testing/overseas?
Other?

<u>ASK ALL:</u>

What are your thoughts about CGP?

Advantages? How do they overcome some of the limitations discussed with standard testing?
Disadvantages?
Thoughts on the report provided?

What type of results are reported?
What features of the overall results do you most value?
How are these results interpreted?
How do you use these results?
Thoughts on time to receive results?

Thoughts on the value of knowing more detailed information on the genetic profile of patients? Is there added value? How so?

E.g. Suitability for specific medication?
E.g. Early opportunity for trial participation?
Other?

To what extent do you believe that CGP should be reimbursed through the Medicare Benefits Schedule (MBS) in Australia?
What do you think is needed to facilitate reimbursement and develop this area in Australia?

</td>
</tr>
<tr>
<td>Understand pathologist’s specific role in gene alteration status testing</td>
<td>4.</td>
<td>
Pathologists Role in Gene Alteration Status Testing (15 min)

Can you take me through the process, and specifically your role in gene alteration status testing for locally advanced (stage IIIb) and metastatic (stage IV) NSCLC?

Who is the key HCP ordering gene alteration status tests? How are tests ordered?
To what extent are you involved in the type of test chosen? How so?

I.e. Do you discuss most suitable test with treating physician (do you make recommendations)? How do you decide on which test to recommend?
Do you advise on the need for additional testing? In what circumstances?

What is involved in the testing process?

Technical aspects with testing? Handling and processing of the specimen (tissue / biopsy)?
How do you run the tests?

Are any tests run overseas? What happens in these circumstances?

</td>
</tr>
</table>

		▪ What is the timeframe for testing and results?<br>o What happens with the test results?<br>▪ Do you send a pathology report to the treating physician?<br>▪ Do you discuss details of results with the treating physician directly? What is discussed?<br>● Do you discuss the accuracy and reliability of the test?<br>● Do you advise on what is clinically relevant and actionable based on the results? How so?<br>● Do you proactively make suggestions to the treating physician on most suitable treatment?<br>o What is your level of involvement with the patient?<br>▪ Patient contact at point of testing? What happens here? Subsequent patient contact?<br>● What do you tell them? Do you discuss test results?<br>● What do they ask / want to know?<br>● To what extent do patients have an awareness and understanding of gene alteration status tests? Specifically, CGP?<br>o To what extent do you feel that patients would value knowing more comprehensive information about their cancer through CGP? Why do you say that?<br>o In your opinion, would there be any disadvantages in patients knowing more comprehensive information through CGP?<br>o What is your role in staying up to date and communicating the latest advancements in gene alteration status testing with other HCPs? How do you feel about this?<br><br><u>Feedback on the role of other HCPs</u><br>To what extent does gene alteration status testing in this area involves a multi-disciplinary approach?<br>● Can you tell me about other HCPs who are involved with newer / more advanced gene alteration status testing (i.e. genomic profiling)?<br>o Type of HCP? Oncologists? Pulmonologists, Radiologists? Nurses? <u>FOR EACH:</u><br>▪ What is their role?<br>▪ How do these HCPs work collaboratively within gene alteration status testing for genomic profiling?<br>● What role do support services have?<br>o Molecular tumour boards?<br>o Advisory services? Which?

<table>
<tr><td></td><td></td><td>Is there anything else you would like to mention about gene alteration status testing that we haven’t already spoken about?</td></tr>
<tr><td>Identify attributes that would be important to pathologists with gene alteration status testing.</td><td>5.</td><td> Perceptions of Attributes Considered Important for Gene Alteration Status Testing (10 min)
As you are aware, this research is to gain feedback on gene alteration status testing for NSCLC.
We have already spoken in detail about these tests. Now I would like to ask you to summarise the key features of gene alteration status testing that are particularly important to you? We will be incorporating the most important features into the design of the next phase of research which is an on-line survey. <u>ALLOW FOR SPONTANEOUS FEEDBACK:</u>
I have a list of features (which we call attributes) that I would like to read to you. I would like to understand how important these are in relation to each other. For each, could you please let me know if you think the attribute is of high, moderate or of low importance to you and why? <u>ONLY ASK ATTRIBUTES AND PROBES THAT HAVE NOT BEEN MENTIONED EARLIER. ASCERTAIN IMPORTANCE OF EACH ATTRIBUTE.</u>
<u>Importance of:</u>

Accuracy of test? How do you assess accuracy?
Test sensitivity? (PROBE ON: diagnostic sensitivity? analytic sensitivity?)
Test specificity?
Mode of biopsy? I.e. what kind of specimen is required?
Tumour vs. liquid (blood test)?
How is this attribute considered during different stages of treatment? (diagnosis, subsequent investigation / repeat testing?)
Reproducibility of test?
Turnaround time for test results?
What do you consider a reasonable timeframe?
Clinical usefulness
Improvement in efficacy of treatment approach selected based on results of gene alteration status test? (E.g. value of knowing about appropriate treatment or non-treatment, reimbursable treatment)
Opportunity to detect an alteration which could result in inclusion in clinical trial?
Type of test? Single marker, hotspot vs. CGP
Value of knowing / level of information obtained?
Size of panel? Large or small?
Ease of result interpretation?
Quality, ease of reading report, clinical relevance (e.g. lists clinical trials, molecular matched therapeutic options)Availability of tumour board?
Risks involved?
What type of risks would you be concerned about?
Where actual GCP takes place?
</td></tr>
</table>

		○ In house vs. send out<br>○ Overseas vs Australia<br>● Ability to claim a fee for providing services associated with testing [e.g. sample preparation, administration (e.g. filling out forms), interpretation of results]<br>● Out of pocket costs? For the test?<br>○ What do you feel would be acceptable costs to patients?<br>● Are there any additional attributes that would be relevant?
Future of gene alteration status testing and wrap up.	6.	**Future of Gene Alteration Status Testing for Locally Advanced (stage IIIb) and Metastatic (stage IV) NSCLC Information** (5 min)<br>What do you believe will be the role of gene alteration status testing for future management and treatment of locally advanced (stage IIIb) and metastatic (stage IV) NSCLC.<br>● What do you think will happen in this area in the next 5 years or so?<br>○ What type of changes are likely? What resources might be required to meet these changes?<br>○ Do you believe the current use of gene alteration status testing will change? (increase / decrease / stay the same?) Why do you say that?<br>▪ What do you believe will drive uptake of gene alteration status tests?<br>▪ What do you believe will drive uptake of genomic testing?<br>● What may drive uptake of use of CGP upfront?<br>▪ How would an increased uptake of genomic testing affect your clinical practice?<br>● Are you aware of innovations that are occurring around gene alteration status testing (In Australia? And overseas?)<br>○ With biopsies? (e.g. collection and interpretation of results?)<br>● What advances with gene alteration status testing would be important to HCPs? And to patients?<br>● Finally, where do you access information for your continued education regarding gene alteration status testing for locally advanced (stage IIIb) and metastatic (stage IV NSCLC?<br>○ How do you stay up to date with developments?<br>● Is there any further information that would be helpful to pathologists / specialists? And to patients?<br>That brings us to the end of our discussion. Thank you for taking the time to participate in this research – it helps with understanding how pathologists are involved with gene alteration status testing for locally advanced (stage IIIb) and metastatic (stage IV) NSCLC. Is there anything else that you would like to add to what we have discussed today?

Supplementary document S2. Value Mapping of Gene Alteration Status Testing Options in Non Small Cell Lung Cancer Among Patients and Clinicians- **Qualitative Patient Discussion Guide**

**Research Objectives (for interviewer reference):**
The aim of this study is to develop a greater understanding of the key attributes / features that people with locally advanced (stage III) and metastatic (stage IV) non-small cell lung (NSCLC) cancer value in their considerations of biomarker testing for their condition.<br>The specific research objectives for this qualitative phase are to enhance understanding of:<br>1. The impact of locally advanced (stage III) and metastatic (stage IV) NSCLC on patients<br>2. Patient awareness and perceptions of biomarker tests<br>3. Key attributes of biomarker testing that are considered valuable by patients and the importance of these (these attributes will be used in the design of the quantitative phase of research).<br>To ensure patient friendly terminology during the interview, locally advanced (stage III) and metastatic (stage IV) lung cancer will be referred to as 'advanced lung cancer' or by the term used by patients themselves (to be explored in the qualitative interview).<br>For similar reasons, recruitment will focus on ALL advanced lung cancer patients (i.e. not specified to NSCLC). While the majority will likely be diagnosed with NSCLC, it is not yet clear how familiar patients are with this terminology and are reluctant to screen-out based on this specific diagnosis.

**Discussion Guide**		
Aim		**Discussion**
To explain purpose of research	1.	**Warn Up and Introduction** (2 min)<br>● *Thank respondent for taking part in the research.*<br>● *Explain purpose of the research being conducted in locally advanced (stage III) and metastatic (stage IV) lung cancer:* Today I would like to talk to you about advanced lung cancer and how this condition has impacted on your life. I am also interested to talk to you about biomarker testing for this condition covering questions such as your awareness of testing and whether you feel results from such testing are valuable for you to know.<br>● *Mention the possibility that the interview may raise questions about their current management of locally advanced (stage III) and metastatic (stage IV) lung cancer:* Participating in this interview may raise questions for you about your health or advanced lung cancer management. If you have any questions, you may wish to discuss them with your doctor.<br>● *Explain anonymity.* Participants are guaranteed under Privacy Act and AMSRS code - findings from interviews will not be linked back to individual people.

		Personal data and responses will not be shared with the sponsor, only aggregated (grouped) data.<br>● *Explain obligation to report Adverse Events (AE):* We are now being asked to pass on to our client, who is a manufacturer of medicines, details of all adverse events related to their own products that are mentioned during the course of market research interviews. Although what you say will, of course, be treated in confidence, should you mention during the discussion an adverse event that you, or someone you know, experienced while taking one of our client's medicines, we will need to report this, so that they can keep the knowledge of the safety profile of their medicines up to date. CONFIRM THAT PARTICIPANT HAS READ AND SIGNED CONSENT FORM<br>● *Ask permission to record (for report-writing purposes).*<br>● *Patient consent:* Are you happy for us to proceed with the interview based on the information you have previously received and which I have just provided to you?
Gain background information on participant, build rapport.	2.	**Participant Profile** (5 min)<br>Can I begin by asking you a little bit about yourself?<br>● Key interests? Age? Work situation? Family / home-life?<br>● Location (rural, regional, metro?)<br>● I would now like to ask you a few background questions about your advanced lung cancer<br>o Firstly, are you treated in a private or public setting for your advanced lung cancer? Are you treated in an out-patient or in-patient setting (or both?)<br>o How long ago were you diagnosed? How was the diagnosis discovered (i.e. what were the events that lead to the diagnosis?)<br>o If you can recall, what is the specific diagnosis you have received for this condition?<br>▪ Are you aware of the stage of your condition?<br>o How do you tend to refer to this condition when you're talking to others?<br>▪ Terminology used with your doctor(s)?<br>▪ Terminology used with family / friends?<br>INTERVIEWER: USE PATIENT'S OWN TERMINOLOGY FOR REMAINDER OF INTERVIEW<br>o Have the following terms ever been used when discussing your condition?<br>▪ Non Small Cell Lung Cancer (NSCLC)<br>▪ Locally advanced (stage IIIb) lung cancer<br>▪ Metastatic (stage IV) lung cancer<br>▪ Advanced / Late stage / Non-operable<br>▪ Adenocarcinoma

		▪ Squamous cell carcinoma<br>▪ Large cell carcinoma<br>▪ Non squamous cell (epidermoid) carcinoma<br>o Do you use any of these terms when talking about your condition?<br>o Have you been diagnosed with any other conditions (in addition to your advanced lung cancer)? Which?
Identify the impact of advanced lung cancer on participant	3.	**Experience of Advanced Lung Cancer** (10 min)<br>Now I would like to ask you about your personal experiences with advanced lung cancer.<br>● What has been the overall impact on your life? How has your life changed?<br>o Physical impact? (INTERVIEWER: BE AWARE OF RELEVANT AE's IF TREATMENT SIDE EFFECTS ARE MENTIONED)<br>o Emotional impact?<br>o Social impact? Impact on relationships?<br>o Impact on family / loved ones? How have their lives changed?<br>o Impact on work?<br>o Financial impact<br>● What are your hopes for the future in relation to your advanced lung cancer? What are your concerns?
Obtain information on awareness and any experiences with biomarker tests for advanced lung cancer	4.	**Awareness and Perceptions of Biomarker Testing for Locally Advanced (stage III) and Metastatic (stage IV) Lung Cancer** (20 min)<br>I'd now like to talk to you about biomarker testing for advanced lung cancer.<br>Patient Awareness<br>● Firstly, are you aware of biomarker testing for advanced lung cancer?<br>o What do you know / have heard about this?<br>o Where did you gain this information?<br>o Has your doctor(s/ any other HCP) talked to you about this? What type of doctor / HCP? What has been discussed?<br>o Are you aware of any specific tests? (FOR INTERVIEWER REFERENCE: EGFR, ALK, ROS, PD-L1?)<br>Are you aware of whether you have undergone biomarker testing in your advanced lung cancer journey?<br>IF SO ASK 'PATIENT BIOMARKER EXPERIENCE' SECTION PRIOR TO READING BIOMARKER INFORMATION:

IF NOT, ASK 'PATIENT BIOMARKER EXPERIENCE' SECTION AFTER READING BIOMARKER INFORMATION

Patient biomarker experience

- Thinking back, for what reason did you receive testing?
- At what point do you believe you have received biomarker tests?
    - At diagnosis? Are you aware of the type of test you received?
        - Tumour tissue biopsy vs. a blood test that examines DNA found in the bloodstream which comes from cancerous cells and tumours (i.e. circulating tumour DNA)
            - IF BLOOD TEST PROBE: Do you recall if a genetic counsellor was involved at all with this blood test ? (INTERVIEWER NOTE: IF SO, LIKELY TO BE A GERMLINE TEST WHEREBY TESTING IS DONE ON NON CANCEROUS CELLS AND THUS FALLS OUTSIDE THE SCOPE OF BIOMARKER TESTS)
        - Number of genes tested?
        - Type of gene tested?
        - Name of test(s)?
    - After diagnosis (i.e. during treatment)? Are you aware of the type of test you received?
        - Tumour tissue biopsy vs. a blood test that examines DNA found in the bloodstream which comes from cancerous cells and tumours (i.e. circulating tumour DNA)
            - IF BLOOD TEST PROBE: Do you recall if a genetic counsellor was involved at all with this blood test? (INTERVIEWER NOTE: IF SO, LIKELY TO BE A GERMLINE TEST WHEREBY TESTING IS DONE ON NON CANCEROUS CELLS AND THUS FALLS OUTSIDE THE SCOPE OF BIOMARKER TESTS)
        - Number of genes tested?
        - Type of gene?
        - Name of test(s)?
    - Upon cancer returning after treatment? Are you aware of the type of test you received?
        - Tumour tissue biopsy vs. a blood test that examines DNA found in the bloodstream which comes from cancerous cells and tumours (i.e. circulating tumour DNA)
            - IF BLOOD TEST PROBE: Do you recall if a genetic counsellor was involved at all with this blood test? (INTERVIEWER NOTE: IF SO, LIKELY TO BE A GERMLINE TEST WHEREBY TESTING IS DONE ON NON CANCEROUS

		CELLS AND THUS FALLS OUTSIDE THE SCOPE OF BIOMARKER TESTS)<br>▪ Number of genes tested?<br>▪ Type of test?<br>▪ Name of test(s)?<br>● What happened in the testing process?<br>● If you are aware, how many times have you received biomarker testing?<br>o Have you had to undergo a re-biopsy of tumour tissue? IF SO: For what reasons? What are your thoughts on this?<br>● How has your treatment choice been impacted, if at all, as a result of testing?<br>● Have you ever been involved in any clinical trials for advanced lung cancer? IF YES: Can you please tell me a little about this? What role did biomarker testing have in this trial? INTERVIEWER: BE AWARE THAT AE'S MAY BE MENTIONED HERE<br>Before we talk about biomarker testing in more detail, I'd like to read some information to you about such tests.<br>READ TO ALL PATIENTS<br>*Your doctor may have discussed having your tumour tissue tested for genetic alterations (or you may have done some research on this yourself). This is sometimes called genetic or genomic testing, biomarker testing, or molecular profiling, and may help you and your doctor make treatment decisions. For example, when some known gene alterations are present in lung cancer cells, some targeted therapies may be more effective than standard chemotherapy.*<br>*Although biomarker testing for some specific genes is standard for all patients with advanced lung cancer at diagnosis, these kinds of tests can be performed at any point during treatment. While these types of tests are usually performed on solid tumour tissue samples, they can sometimes be done by isolating circulating tumour cells or DNA in a blood sample.*<br>*There are different types of biomarker tests available. Standard genetic tests involve testing one gene at a time across a pre-specified region of the cancer cells' DNA. Newer tests can examine 10's to 1000's of genes at the same time using a DNA sequencing technology called Next Generation Sequencing (NGS). NGS can be used to target specific regions of known cancer genes that have been well characterised as mutational hotspots (i.e. hotspot panel tests) and also for Comprehensive Genomic Profiling (CGP) which looks for alterations in the entire coding region of hundreds of genes known to drive cancer.*<br>GO BACK AND ASK ABOUT 'PATIENT BIOMARKER TEST EXPERIENCE' SECTION IF NOT ASK PREVIOUSLY<br>ASK ALL:

Patient Terminology

- How do you (or would you) refer to these tests if you were talking about them to others? What name do / would you use for them?
  - What name (or term) do you / would you use with your doctor? What does your doctor call them?
  - What name (or term) do you / would you use with your family / friends?
  - What other names have you heard (if any) for these tests? PROBE:
    - Genomic Sequencing?
    - Genetic testing?
    - Mutation testing?
    - Molecular testing?

HCP responsible for decisions relating to biomarker testing

- Who has been the key healthcare professional (HCP) involved in biomarker testing? (E.g. Oncologist?)
- Can you tell me about discussion you have had about biomarker testing with your oncologist?
  - What has been discussed? (i.e. reason for test?)
  - When have these discussions occurred (at diagnosis, during treatment, remission etc)?
  - To what extent have you been involved in decisions relating to biomarker testing for your condition? E.g. decision to undergo testing, the type of test to have etc?
- Have there been any other HCPs that have been involved in testing?
  - GP?
  - Pathologist?
  - Nurse?
  - Other?
- Are you aware of any communication between your oncologist and other HCPs regarding biomarker testing? Which type?
  - Pathologists? Are you aware of what is discussed at all?

Overall perceptions of biomarker tests

Based on what we have just discussed and the information on biomarker tests that I read to you earlier, what are your overall thoughts on such tests for advanced lung cancer?

- Perceived positives? How do you feel you could benefit from testing, if at all?
- Perceived negatives? What are your concerns, if any?

		○ How could these concerns be alleviated? (e.g. provision of information - what type?)<br>● To what extent are you willing to undergo biomarker tests? Why do you say that?<br>● How willing would you be to change your current therapy based on test results?<br><br><u>Preferred tests</u><br>*As mentioned earlier, current standard of care for biomarker tests involve single-gene tests which investigate specific genes, one at a time - these specific genes have reimbursed treatments available.*<br><br>*The newer tests which include comprehensive genomic profiling (CGP) provide a more detailed picture of your cancer by looking at multiple gene alterations, some of which have reimbursable treatments. However, these newer tests are not currently reimbursed, and some gene alterations may be linked to treatment options that are not reimbursed to patients and are only available in clinical trials or by private purchase. Additional information can also be obtained from these tests for which there is no current treatments available.*<br><br>I am interested to hear about the type of tests you would prefer and the reasons for this?<br>● If you were responsible for choosing a biomarker test, what type would you choose and why?<br>○ Tumour tissue biopsy vs. liquid biopsy (blood test)? Why do you say that?<br>▪ What are the advantages / disadvantages of a tumour tissue biopsy and advantages / disadvantages of liquid biopsy (blood test)?<br>○ Single genetic tests or genomic tests (test providing a more detailed picture)? Why do you say that?<br>▪ What are the advantages / disadvantages of a single test and the advantages / disadvantages of genomic tests?<br><br><u>Further thoughts on CGP</u><br>Just a few specific questions on CGP (this test provides information on over 300 genes)<br>● To what extent do you desire such detailed information on the profile of your advanced lung cancer rather than testing only for one gene at a time (as with standard tests)? Why do you say that?<br>○ What is the value to you of knowing a detailed picture?<br>▪ E.g. higher predictability of outcome? greater confidence in treatment plan?

		▪ The test itself is not currently being reimbursed – would you be willing to pay out of pocket?<br>○ What is the value of knowing a detailed picture if the full information may not lead to any direct benefit to you? <u>PROBE:</u> For example:<br>▪ Knowing there is treatment available that could be beneficial, however is not reimbursed (i.e. you would have to pay for this treatment yourself). How would you feel about this?<br>● Would you consider paying privately?<br>● As an estimate, how much would you be willing to spend per month for a non-reimbursed treatment?<br>▪ Knowing there is treatment which may be beneficial which is still under investigation in clinical trials?<br>● To what extent would you value information that would lead to a higher probability of being included in a clinical trial?<br>● To what extent would you wish to participate in clinical trials in this circumstance?<br>▪ Knowing that your HCP has utilised all options to find an additional treatment or that you are currently treated with optimal treatment?<br>○ Would there be any disadvantages in knowing a detailed picture through CGP?<br>▪ What type? (e.g. uncertain, unanticipated or uninformative information; emotional concerns / anxiety)<br>○ At what point would you want to receive CGP? [i.e. upfront (i.e. at diagnosis) or later in the treatment journey? Why do you say that?]
Identify attributes that would be important with biomarker *testing.*	5.	**Perceptions of Biomarker Testing / Attributes Considered Important** (20 min)<br>Part of this research is to understand what aspects of biomarker testing are important to people with advanced lung cancer in their choice of a biomarker test.<br>If you were to receive biomarker testing for advanced lung cancer, what features of this testing would be particularly important to you? Why these? <u>PROBE FULLY</u><br>I have a list of features (which we call attributes) that may or may not be important to you with biomarker testing for advanced lung cancer. I would like to read these to you and obtain your thoughts on how important you regard each of these attributes. For each, could you please let me know if you think the attribute is of high, moderate or low importance to you and your reasons for this.<br><u>DISCUSS EACH ATTRIBUTE WITH PARTICIPANT. CAPTURE WHETHER OF HIGH, MODERATE OR LOW IMPORTANCE</u><br>● Accuracy of test? What things about a test makes you think it is accurate?

<table>
<tr><td></td><td></td><td>● Type of biopsy? I.e. what kind of sample is required?
    o Tumour tissue vs. liquid (blood test)?
    o How may this vary at different stages of your treatment? (e.g. diagnosis, further investigation / repeat testing?)
● Type of test? Single marker, hotspot vs. CGP?
    o Value of knowing / level of information obtained?
● Size of panel? Large or small?
● Reproducibility of test? (i.e. to measure any changes in your condition)
● Time to receive test results?
    o What do you consider a reasonable timeframe?
● How useful a test is?
    o E.g. in guiding treatment decisions (E.g. whether you are likely to benefit from a particular therapy?)
● Risks involved?
    o What type of risks would you be concerned about?
● Out of pocket costs?
    o For the test? What type of costs would you be willing to accept?
● Where the actual GCP/testing takes place?
    o Overseas vs Australia</td></tr>
<tr><td>Feedback on advanced lung cancer information and wrap up.</td><td>6.</td><td>Feedback on Access to Advanced Lung Cancer Information (3 min)
● Finally, I'd just like to ask you from where you have gained information and an understanding about your advanced lung cancer?
    o And specifically, regarding biomarker tests?
● Are you aware of any new advancements (treatment and biomarker tests) becoming available for advanced lung cancer? <u>IF SO</u>: What have you heard? Where have you heard this?
● Is there anything else that would be helpful to you regarding further information about biomarker tests?
That brings us to the end of our discussion. Thank you for taking the time to participate in this research – it helps with understanding your perspective on advanced lung cancer. Is there anything else that you would like to add to what we have discussed today?</td></tr>
</table>

Supplementary document S3. Value Mapping of Gene Alteration Status Testing Options in Advanced Non Small Cell Lung Cancer Among Patients and Clinicians-**Qualitative Oncologist Discussion Guide**

**Research Objectives (for interviewer reference):**
The aim of this study is to develop a greater understanding of the key attributes / features that oncologists consider in their selection of gene alteration status tests for patients with locally advanced (stage IIIb) and metastatic (stage IV) Non Small Cell Lung Cancer (NSCLC).<br><br>The specific research objectives for this qualitative phase are to enhance understanding of:<br><br>1. Oncologist use and perceptions of gene alteration status testing within locally advanced (stage IIIb) and metastatic (stage IV) NSCLC.<br>2. What factors oncologists consider in deciding to order a gene alteration status test for this condition and once a decision has been made, what factors influence oncologists' choice for a specific test.<br>3. Key attributes of gene alteration status testing that are considered valuable by oncologists and the relative importance of these (attributes discussed in the qualitative research will be used in the design of the quantitative survey)<br>4. What may drive uptake of these tests<br><br>FOR INTERVIEWER REFERENCE:<br>**For this research, tests that fall within the scope of 'gene alteration status testing' include:**<br>1) Any assays that detect somatic alterations in solid tissue or liquid biopsy [e.g. detection of circulating DNA (cDNA) or circulating tumour DNA (ctDNA)]; that is alterations that occur spontaneously over the course of a person's lifetime or through interaction with known carcinogens.<br>2) Any standard diagnostic (e.g. IHC/FISH) detecting qualitative sequence level changes (e.g. EGFR mutations variants or alterations,) or quantitative expression level (protein) biomarkers such as (e.g. PD-L1)<br>3) Any large or small gene panel using next-generation sequencing (NGS) technology with an assay based on PCR or amplicon (e.g. Hotspot) or hybrid-capture [e.g. Comprehensive genomic profiling (CGP)] target enrichment.<br>4) Whole exome or whole genome sequencing performed on DNA extracted from tumour tissue.<br><br>**For this research, tests that fall outside the scope of 'gene alteration status testing' include:**<br>1) Any assay that detects germline alterations; that is alterations that are inherited and occur in families (e.g. BRCA1 and BRCA2). These tests would be done in DNA extracted white blood cells in peripheral whole blood and a genetic counsellor would be involved to counsel the patient and their family members.

**Discussion Guide**		
Aim		**Discussion**
To explain purpose of research	1.	**Warm Up and Introduction** (2 min)<br>● Thank respondent for taking part in the research.

		● *Explain purpose of the research:* We are conducting research in the area of locally advanced (stage IIIb) and metastatic (stage IV) NSCLC. I am interested to understand your perceptions and use of gene alteration status testing for patients with this condition.<br><br>● *Explain anonymity:* Participants are guaranteed under Privacy Act and AMSRS code - findings from interviews will not be linked back to individual people. Personal data and responses will not be shared with the sponsor of this research, only aggregated data.<br>● *Explain obligation to report Adverse Events (AE):* Although all responses will, of course, be treated in confidence, should you raise an adverse event experienced by a patient after using one of our client's products or medications, we will need to report this, so that they can keep the knowledge of the safety profile of their medicines up to date. This also applies if the event has already been reported by you directly to the company or the regulatory authority <u>CONFIRM THAT DR HAS READ AND SIGNED CONSENT FORM</u><br>● *Ask permission to record (for report-writing purposes).*
Gain background information on participant.	2.	**Participant Profile** (3 min)<br>Can I begin by asking you a little about your work situation?<br>● Where is your key place of work?<br>o Proportion in each of private / public settings?<br>o Location? (Rural, regional, metro?)<br>o Socio-economic area?<br>● Can you please summarise what your key duties / responsibilities are?<br>● How does locally advanced (stage IIIb) and metastatic (stage IV) NSCLC fit into your workload?<br>● Roughly, what proportion of your overall patient workload is for locally advanced (stage IIIb) and metastatic (stage IV) NSCLC?
Gain feedback on terminology for locally advanced (stage III) and metastatic (stage IV) NSCLC.	3.	**Background on Locally advanced (stage IIIb) and Metastatic (stage IV) NSCLC Patients** (5 min)<br>Thinking about locally advanced (stage IIIb) and metastatic (stage IV) NSCLC.<br>● What terminology do you personally use when referring to this condition?<br>o With other HCPs?<br>o With patients?<br>● How do patients refer to this condition? (i.e. NSCLC, lung cancer?)<br>o Do they tend to be aware of their specific diagnosis?<br>o Do they tend to be aware of the staging of their condition?<br>o Do they tend to be aware that their cancer is inoperable?<br>● Can you summarise what is involved in terms of the histology and staging for diagnosis of this condition.

<table>
<tr>
<td>Determine use and perceptions of gene alteration status testing.</td>
<td>4.</td>
<td>
Perceptions and Use of Gene Alteration Status Testing (30 min)
I’d now like to talk to you about your perceptions and experience with gene alteration status testing for patients with locally advanced (stage IIIb) and metastatic (stage IV) NSCLC.
For the purpose of this research, tests that fall within the scope of ‘gene alteration status testing’ include:
1) Any assays that detect somatic alterations in solid tissue or liquid biopsy [e.g. detection of circulating DNA (cDNA) or circulating tumour DNA (ctDNA)]; that is alterations that occur spontaneously over the course of a person’s lifetime or through interaction with known carcinogens.
2) Any standard diagnostic (e.g. IHC/FISH) detecting qualitative sequence level changes (e.g. EGFR mutations variants or alterations,) or quantitative expression level (protein) biomarkers such as (e.g. PD-L1)
3) Any large or small gene panel using next-generation sequencing (NGS) technology with an assay based on PCR or amplicon (e.g. Hotspot) or hybrid-capture [e.g. Comprehensive genomic profiling (CGP)] target enrichment.
4) Whole exome or whole genome sequencing performed on DNA extracted from tumour tissue.
Tests that fall outside the scope of ‘gene alteration status testing’ include:
1) Any assay that detects germline alterations; that is alterations that are inherited and occur in families (e.g. BRCA1 and BRCA2). These tests would be done in DNA extracted white blood cells in peripheral whole blood and a genetic counsellor would be involved to counsel the patient and their family members.
<u>Terminology Used</u>

Firstly, what terminology do you use when referring to such tests?

When talking to other HCPs?
When talking to patients?

Does terminology vary depending on what type of test is ordered? How so?

<u>USE PARTICPANT’S TERMINOLOGY FOR THE REMAINDER OF INTERVIEW</u>
<u>General Perceptions of gene alteration status testing</u>

What do you believe is the key role of gene alteration status testing for locally advanced (stage IIIb) and metastatic (stage IV) NSCLC?

What are the reasons gene alteration status tests are used in this therapy area?

What are the positives with gene alteration status testing? How can patients benefit?
What are challenges in the current (single) gene alteration status testing landscape? <u>POTENTIAL PROBES:</u>

</td>
</tr>
</table>

- Biopsy sampling? (e.g. limited tumour tissue?, Preservation of tissue?, Need for re-biopsy?)
    - Thoughts on tissue vs. liquid biopsies? Which do you prefer and why?
- Accuracy of test?
    - How do you assess accuracy? Do you have a certain standard or minimum accuracy?
    - Can you tell me about ‘false negatives’ and ‘false positives with a test? How does this impact on patients?
- Interpretation of results? Who has the key responsibility for this? Oncologists? Other HCP?
- Turn around for test results? How so? What does this mean for patients?
- A general lack of testing awareness and understanding?
    - Among HCPs? Among patients?
- Risks involved? What type of risks?
- Legislation issues?
- Patient consent? How so?

- What factors do you consider in deciding to order a gene alteration status test for a patient?
- And then, what are the key factors you consider in choosing a specific test (e.g. accuracy, time to test results, test or treatment reimbursement factors)?

I’d now like to ask you about different gene alteration status tests used for locally advanced (stage IIIb) and metastatic (stage IV) NSCLC.

<u>Feedback on conventional / standard gene alteration status algorithm</u>

- Can you please take me through the standard gene alteration status testing algorithm for this condition? I.e. sequential single marker genetic tests? <u>PROBE ON</u>:
    - What tests are used and why? (e.g. EGFR, ALK, ROS?)
    - How do you use these tests?
        - At what point in the patient pathway (i.e. at diagnosis of advanced / metastatic disease? during treatment journey?)
        - Typically, how often are you ordering these tests for an individual patient?
    - How do these tests guide treatment decisions?
    - What are your overall thoughts about standard testing?
        - Advantages - what type of concrete improvements do they provide for patients?
        - Disadvantages? Limitations?

Feedback on advancements in gene alteration status testing

- Can you now tell me about newer / more advanced gene alteration status testing options within locally advanced (stage IIIb) and metastatic (stage IV) NSCLC? I.e. Genomic Profiling?
    - Next Generation Sequencing (NGS)
        - Hot Spot Panels
        - Comprehensive Genomic Profiling (CGP)
    - Other?
- How do these tests fit in your clinical practice?

Feedback on Comprehensive Genomic Profiling (CGP)

I'd now like to focus on CGP.

- Have you had experience using CGP for your patients? IF YES, ASK:
    - In what type of setting? Public vs. private?
    - What proportion of these are via tumour tissue vs. liquid biopsy panels?
- For what reasons do you decide to specifically order CGP tests (as opposed to standard tests)? In what situations do you order these?
    - Patient requests?
    - Patient factors? Do you use any assessment tools? [e.g. ECOG (Eastern Co-operative Oncology Group) Scale of Performance Status, Other?] How so?
    - When requiring additional insight vs. single marker tests?
- At what point in the patient pathway (i.e. before 1st-line treatment?; Progressive disease and lack of patient response?) FOR PROGRESSIVE DISEASE / LACK OF PATIENT RESPONSE
    - Would you have preferred to do this upfront after diagnosis? Why do you say that?
- How often are you ordering these tests for an individual patient?
- How do these tests guide treatment decisions?
    - How do they differ in this regard compared to standard tests?
- How may a treatment change as a result of CGP tests, if at all?
- Can you provide examples of the specific CGP tests you are ordering?
    - Lab developed tests/inhouse testing?
    - Send out testing/overseas?
    - Other?

ASK ALL:

- What are your thoughts about CGP?
    - Advantages? How do they overcome some of the limitations discussed with standard testing?
    - Disadvantages?
    - Thoughts on the report provided?
        - What type of results are reported?
        - What features of the overall results do you most value?

        - How are these results interpreted?
        - How do you use these results?
        - Thoughts on time to receive results?
    - Thoughts on the value of knowing more detailed information on the genomic profile of your patient's NSCLC? Is there added value? How so?
        - E.g. Suitability for specific medication?
        - E.g. Early opportunity for trial participation?
        - Other?
    - To what extent do you believe that CGP should be reimbursed through the Medicare Benefits Schedule (MBS) in Australia?
    - What do you think is needed to facilitate reimbursement and develop this area in Australia?

Now, I would just like to ask a few more questions about gene alteration status testing for locally advanced (stage IIIb) and metastatic (stage IV) NSCLC:

<u>Preferred testing method for gene alteration status</u>

Can you tell me about the tissue you prefer to use to test for gene alteration status / genomic alterations?

- Initial biopsy?
    - E.g. biopsy from first diagnosis / at determination of locally advanced (stage IIIb) and metastatic (stage IV) disease?
- Re-biopsy?
    - Biopsy from primary tumour tissue?
    - Biopsy from potential metastases?
- Are you aware of what proportion of patients require re-biopsy due to insufficient sampling of initial biopsy?
- At what point would you do a re-biopsy (initially or later in pathway)
- What are your thoughts on having to re-biopsy a patient?

<u>Feedback on patient awareness of gene alteration status tests</u>

- Typically, what level of awareness do patients have regarding gene alteration status testing?
    - Are they aware of gene alteration status tests at all? Do they know if they have received gene alteration status testing?
    - How much do they understand about testing? I.e. Reasons for testing?
    - Do they know about different tests? (e.g. would they know specifically about CGP? Would they know about specific brands of CGP?)
    - What are their perceptions of these tests (perceived benefits / challenges)?

		● To what extent do you talk to patients about gene alteration status testing? What is discussed? When is this discussed in the patient journey?<br>o What do you tell them?<br>o What do they ask / want to know?<br>o In the scheme of things, how interested are they in gene alteration status tests?<br>● To what extent are they involved in decisions regarding gene alteration status testing (e.g. which test is ordered?)<br>● And specifically, in terms of CGP, to what extent do you feel that patients would value knowing more comprehensive information about their cancer through CGP?<br>o What would be valuable to them? (e.g. higher predictability of outcome? greater confidence in treatment plan?)<br>o What would be the value for patients of knowing comprehensive information if it may not lead to any direct clinical benefit? <u>PROBE:</u><br>▪ E.g. Knowing there is treatment available that could be beneficial however is not reimbursed?<br>● Do you feel patients would consider paying privately?<br>▪ E.g. Knowing there is treatment which may be beneficial which is still under investigation in clinical trials?<br>▪ E.g. Knowing that they have tried all available treatment options or that treatment has already been optimised<br>o To what extent do you feel that there would be disadvantages for patients in knowing a comprehensive picture through CGP?<br>▪ What type? (e.g. uncertain, unanticipated or uninformative information; emotional concerns / anxiety for patients)<br><u>Feedback on the role of pathologists and other HCPs</u><br>Now I would like to ask you about the extent to which gene alteration status testing in this area involves a multi-disciplinary approach?<br>● Can you tell me about other HCPs who are involved with newer / more advanced gene alteration status testing (i.e. genomic profiling)?<br>o Type of HCP? Pathologists? Pulmonologists, Radiologists? Nurses? <u>FOR EACH:</u><br>▪ What is their role?<br>▪ How do these HCPs work collaboratively within gene alteration status testing for advanced NSCLC?<br><u>IF NOT COVERED SPONTANEOUSLY</u>:<br>● What role do pathologists have?

		o Do they advise on what test to order?<br>o What is their involvement in interpreting results? How does this impact on treatment?<br>▪ How important are pathologists to the quality of the outcome?<br>▪ To what extent do pathologists provide advice on what is clinically relevant and actionable based on the results?<br>● What role do support services have?<br>o Molecular tumour boards?<br>o Advisory services? Which?<br><u>Differences existing with gene alteration status testing within various settings</u><br>And now, just a few questions regarding use of gene alteration status testing in different areas or settings within locally advanced (stage IIIb) and metastatic (stage IV) NSCLC.<br>● To what extent are there differences in use of gene alteration status tests between states or in metro vs. regional and rural areas (if any)?<br>o How does this impact on patient management in metro vs. regional and rural areas?<br>● To what extent are there differences in the private vs. public setting (if any)?<br>o How does this impact on patient management in private vs. public setting?<br>● Is there anything else you would like to mention about gene alteration status testing that we haven't already spoken about?
Identify attributes that would be important with gene alteration status testing.	5.	**Perceptions of Attributes Considered Important for Gene Alteration Status Testing** (10 min)<br>As you are aware, this research is to gain feedback on gene alteration status testing for NSCLC.<br>We have already spoken in detail about these tests. Now I would like to ask you to summarise the key features of gene alteration status testing that are particularly important to you? We will be incorporating the most important features into the design of the next phase of research which is an on-line survey. <u>ALLOW FOR SPONTANEOUS FEEDBACK:</u><br>I have a list of features (which we call attributes) that I would like to read to you. I would like to understand how important these are in relation to each other. For each, could you please let me know if you think the attribute is of high, moderate or of low importance to you and why? <u>ONLY ASK ABOUT ATTRIBUTES THAT HAVE NOT BEEN MENTIONED SPONTANEOUSLY. ASCERTAIN IMPORTANCE OF EACH ATTRIBUTE.</u>

<table>
<tr><td></td><td></td><td>

- Accuracy of test? How do you assess accuracy?
  - Test sensitivity? (PROBE ON: diagnostic sensitivity? analytic sensitivity?)
  - Test specificity?
- Mode of biopsy? I.e. what kind of specimen is required?
  - Tumour vs. liquid (blood test)?
  - How is this attribute considered during different stages of treatment? (diagnosis, subsequent investigation / repeat testing?)
- Reproducibility of test?
- Turnaround time for test results?
  - What do you consider a reasonable timeframe?
- Clinical usefulness
  - Improvement in efficacy of treatment approach selected based on results of gene alteration status test? (E.g. value of knowing about appropriate treatment or non-treatment, reimbursable treatment)
  - Opportunity to detect an alteration which could result in inclusion in clinical trial?
- Type of test? Single marker, hotspot vs. CGP
  - Value of knowing / level of information obtained?
- Size of panel? Large or small?
- Ease of result interpretation?
  - Quality, ease of reading report, clinical relevance (e.g. lists clinical trials, molecular matched therapeutic options)?
  - Availability of tumour board?
- Risks involved?
  - What type of risks would you be concerned about?
- Where actual CGP takes place?
  - In house vs. send out
  - Overseas vs Australia
- Ability to claim a fee for providing services associated with testing (e.g. sample preparation, administration (e.g. filling out forms), interpretation of results)
- Out of pocket costs? For the test?
  - What do you feel would be acceptable costs to patients?
- Are there any additional attributes that would be relevant?

</td></tr>
<tr><td>

Future of gene alteration status testing and wrap up.

</td><td>7.</td><td>

Future of Gene Alteration Status Testing for Locally Advanced (stage III) and Metastatic (stage IV) NSCLC (5 min)

I'd like to ask you what you believe will be the role of gene alteration status testing for future management and treatment of locally advanced (stage IIIb) and metastatic (stage IV) NSCLC.

- What do you think will happen in this area in the next 5 years or so?
  - What type of changes are likely? What resources might be required to meet these changes?
  - Do you believe the current use of gene alteration status testing will change? (increase / decrease / stay the same?) Why do you say that?

</td></tr>
</table>

        - What do you believe will drive uptake of gene alteration status tests?
        - What do you believe will drive uptake of genomic testing?
            - What may drive uptake of use of CGP upfront?
        - How would an increased uptake of genomic testing affect your clinical practice?
- Are you aware of innovations that are occurring around gene alteration status testing (In Australia? And overseas?)
    - With biopsies? (e.g. collection and interpretation of results?)
- What advances with gene alteration status testing would be important to HCPs? And to patients?

- Finally, where do you access information for your continued education regarding gene alteration status testing?
- Is there any further information that would be helpful to oncologists? And to patients?

That brings us to the end of our discussion. Thank you for taking the time to participate in this research – it helps with understanding how specialists regard gene alteration status testing for locally advanced (stage IIIb) and metastatic (stage IV) NSCLC. Is there anything else that you would like to add to what we have discussed today?
